# Genomic prediction of regional-scale performance in switchgrass (*Panicum virgatum*) by accounting for genotype-by-environment variation and yield surrogate traits

**DOI:** 10.1093/g3journal/jkae159

**Published:** 2024-07-19

**Authors:** Neal W Tilhou, Jason Bonnette, Arvid R Boe, Philip A Fay, Felix B Fritschi, Robert B Mitchell, Francis M Rouquette, Yanqi Wu, Julie D Jastrow, Michael Ricketts, Shelley D Maher, Thomas E Juenger, David B Lowry

**Affiliations:** Department of Plant Biology, Michigan State University, East Lansing, MI 48824, USA; Department of Integrative Biology, University of Texas at Austin, Austin, TX 78712, USA; Department of Agronomy, Horticulture and Plant Science, South Dakota State University, Brookings, SD 57006, USA; Grassland, Soil and Water Research Laboratory, USDA-ARS, Temple, TX 76502, USA; Division of Plant Science & Technology, University of Missouri, Columbia, MO 65201, USA; Wheat, Sorghum, and Forage Research Unit, USDA-ARS, Lincoln, NE 68583, USA; Texas A&M AgriLife Research and Extension Center, Texas A&M University, Overton, TX 75682, USA; Department of Plant and Soil Sciences, Oklahoma State University, Stillwater, OK 74075, USA; Environmental Science Division, Argonne National Laboratory, Lemont, IL 60439, USA; Environmental Science Division, Argonne National Laboratory, Lemont, IL 60439, USA; USDA-NRCS, E. “Kika” de la Garza Plant Materials Center, Kingsville, TX 78363, USA; Department of Integrative Biology, University of Texas at Austin, Austin, TX 78712, USA; Department of Plant Biology, Michigan State University, East Lansing, MI 48824, USA; Great Lake Bioenergy Research Center, Michigan State University, East Lansing, MI 48824, USA; Plant Resilience Institute, Michigan State University, East Lansing, MI 48824, USA

**Keywords:** switchgrass, climate change, genomic prediction, genotype by environment

## Abstract

Switchgrass is a potential crop for bioenergy or carbon capture schemes, but further yield improvements through selective breeding are needed to encourage commercialization. To identify promising switchgrass germplasm for future breeding efforts, we conducted multisite and multitrait genomic prediction with a diversity panel of 630 genotypes from 4 switchgrass subpopulations (Gulf, Midwest, Coastal, and Texas), which were measured for spaced plant biomass yield across 10 sites. Our study focused on the use of genomic prediction to share information among traits and environments. Specifically, we evaluated the predictive ability of cross-validation (CV) schemes using only genetic data and the training set (cross-validation 1: CV1), a subset of the sites (cross-validation 2: CV2), and/or with 2 yield surrogates (flowering time and fall plant height). We found that genotype-by-environment interactions were largely due to the north–south distribution of sites. The genetic correlations between the yield surrogates and the biomass yield were generally positive (mean height *r* = 0.85; mean flowering time *r* = 0.45) and did not vary due to subpopulation or growing region (North, Middle, or South). Genomic prediction models had CV predictive abilities of −0.02 for individuals using only genetic data (CV1), but 0.55, 0.69, 0.76, 0.81, and 0.84 for individuals with biomass performance data from 1, 2, 3, 4, and 5 sites included in the training data (CV2), respectively. To simulate a resource-limited breeding program, we determined the predictive ability of models provided with the following: 1 site observation of flowering time (0.39); 1 site observation of flowering time and fall height (0.51); 1 site observation of fall height (0.52); 1 site observation of biomass (0.55); and 5 site observations of biomass yield (0.84). The ability to share information at a regional scale is very encouraging, but further research is required to accurately translate spaced plant biomass to commercial-scale sward biomass performance.

## Introduction

Climate change will be expensive, catastrophic, or both (Masson-Delmotte *et al*. 2021). One strategy to mitigate historical and ongoing carbon emissions is through direct air capture of excess atmospheric carbon (Hanna *et al*. 2021). Agriculture and forestry are currently the most scalable approaches for direct air capture (Azar *et al*. 2010; Turner *et al*. 2018). Once captured, carbon in biomass can be used as a carbon neutral energy source, and a portion of emissions can then be processed and transferred into long-term geologic storage (Fajardy and Dowell 2017; Gelfand *et al*. 2020). Alternatively, if energy costs are prohibitively low, and trustworthy carbon offset markets are established, biomass carbon can be processed to exclusively target carbon sequestration (Woodall and McCormick 2022; Pett-Ridge *et al.* 2023). While efficient long-term carbon capture using biomass is now becoming more feasible, it will require a resource-efficient production of biomass at a meaningful scale, which will require the improvement of biomass crops through future breeding efforts.

Switchgrass is a long-lived warm-season grass and a promising biomass crop, which is native to the central and eastern United States as well as regions of Mexico and Canada (Casler *et al*. 2018b; Lowry *et al*. 2019). Switchgrass can produce large quantities of biomass, is drought tolerant and highly nutrient efficient, and may promote soil carbon accumulation (Cassida *et al*. 2005; Liebig *et al*. 2008; Fike *et al*. 2017; Casler *et al*. 2018b; Lovell *et al*. 2021). Perennial grass biomass through plants like switchgrass is also labor and fuel efficient since established fields require few inputs during the growing season, and a single harvest can occur between August and the following May (Schmer *et al*. 2008; Sadeghpour *et al*. 2014; Serapiglia *et al*. 2016; Casler 2022). In addition, there is evidence that root-associated diazotroph communities can fix atmospheric nitrogen to support switchgrass productivity (Roley *et al.* 2018, 2021). This reduces the use of energy-intensive nitrogen fertilizer. Despite these benefits, robust biomass markets have not developed, preventing grower adoption of switchgrass as a crop and preventing innovation associated with an active industry (Perrin *et al*. 2008; Namoi *et al*. 2022). Agronomically, the most promising route to improved adoption is through the release of higher yielding cultivars (Casler *et al*. 2018; Namoi *et al*. 2022).

Switchgrass breeding progress is limited by its complex reproduction and time-consuming field evaluations. It is strongly out-crossing, and breeders are limited to family-based evaluations (Martínez-Reyna and Vogel 2002; Casler and van Santen 2010; Liu and Wu 2012). Furthermore, it is a long-lived perennial, which can require 3–5 years of field data to obtain reliable performance estimates in commercial-scale swards (Sykes *et al*. 2016). Switchgrass cultivars also have strong genotype-by-environment (GxE) interactions (McMillan 1959; Liebig *et al.* 2008; Casler 2012; Casler *et al*. 2019; Napier *et al*. 2022). They are generally adapted to regions within 1–2 hardiness zones from their original collection locations (Vogel *et al*. 2005; Casler *et al*. 2007). Despite these limitations, the switchgrass breeding progress for increased biomass yield has been between 1 and 4% per year, which is comparable to maize (1.0–1.5% per year) and wheat (0.5–1.5% per year) despite a large disparity in breeding investment (Duvick 2005; Schmidt 1984; Green *et al*. 2012; Casler and Vogel 2014).

Recent advances can accelerate breeding progress in switchgrass. Multiple studies have reported promising yield surrogates, which are traits that can be measured on individual plants and are correlated with the biomass yield (Price and Casler 2014: fall height and flowering time; Sykes *et al*. 2017: fall height; Tilhou and Casler 2022a: flowering time). Reliable yield surrogates can allow a rapid selection of superior individuals, instead of families, and can shorten breeding cycles (Casler and Brummer 2008). To be valuable, a surrogate measure must have greater heritability than biomass yield measurements and a strong genetic correlation with biomass yield. In addition, it is beneficial for the measurement to be less expensive or require fewer years of evaluation.

The most promising switchgrass yield surrogate is flowering time, with delayed flowering being highly positively correlated with the biomass yield (Casler 2020; Tilhou and Casler 2022a). Flowering time can be easily measured on individual plants and is almost entirely controlled by a combination of photoperiod and heat units, which results in reliable GxE effects (Schwartz and Amasino 2013; Sarath *et al*. 2014). These results, however, have only been reported for the North-Central United States, where harsh winters have forced breeders to focus on germplasm containing the early flowering Midwest subpopulations of switchgrass (largely equivalent to the upland ecotype; Casler *et al*. 2018; Poudel *et al*. 2020; Lovell *et al*. 2021). Midwest subpopulations flower and begin senescence months before frost terminates potential growth. Therefore, the selection for later flowering extends the vegetative biomass accumulation in Midwest subpopulations with seemingly no negative repercussions. The applicability of this research to the southern or mid-southern United States is unknown since the preferred germplasm source in the southern United States is the late flowering lowland ecotype, which includes the Gulf and Texas subpopulations. It is reasonable to assume that delayed flowering is not as valuable of a surrogate for biomass in these already late flowering populations, but this has not been evaluated.

Genomic prediction is a second strategy that can accelerate yield improvement. Genomic prediction improves estimates of breeding values for genotypes in a field trial by linking field performance directly to relationships among genotypes based on a large number of DNA markers (Meuwissen *et al*. 2001). In switchgrass, this has improved the accuracy for both sward biomass yield and yield surrogates (Lipka *et al*. 2014; Casler and Ramstein 2018; Fiedler *et al*. 2018; Poudel *et al*. 2019; Tilhou and Casler 2022a). Ramstein and Casler (2019) reported that a successful implementation of the genomic selection could increase switchgrass breeding progress 2–3-fold relative to traditional family evaluation and selection.

Genomic prediction has revolutionized breeding programs by allowing information to be shared among genotyped individuals and among individuals in different growing environments (Heffner *et al*. 2010; Crossa *et al*. 2017). For plant breeders, this facilitates sparse experimental designs, which use genomic data to share information among many environments with minimal replication of genetic units within an environment (Endelman *et al*. 2014; Atanda *et al*. 2021; Montesinos-Lopez *et al*. 2022). The optimum degree of genotype replication is an important variable and dependent on growing environment variability, genotyping resources, and trait heritability (González-Barrios *et al*. 2019). In the most extreme case, the performance of new genotypes can be predicted through a cross-validation (CV) scheme using only genetic data (cross-validation 1: CV1; Burgueño *et al*. 2012; Jarquín *et al*. 2017). Alternatively, genotypes can be evaluated in a subset of environments (cross-validation 2: CV2), which is a strategy that is often used when seed is scarce. Further improvements in the prediction accuracy have been reported by supplementing multisite evaluations with multitrait evaluations (Fernandes *et al*. 2018; Bhatta *et al*. 2020; Lenz *et al*. 2020; Gill *et al*. 2021). Multitrait models use trait covariates or surrogates to improve model precision for an otherwise challenging breeding target (Edmé and Mitchell 2021). Multitrait and multienvironment models are the most valuable when a breeding target trait cannot be economically evaluated in all environments and when included traits have strong genetic correlations.

This study examined several topics related to breeding switchgrass using an ongoing common garden experiment that evaluated a diverse set of 630 switchgrass genotypes from 4 major switchgrass subpopulations across 10 different sites for 3 years (Lovell *et al*. 2021). There were 4 objectives of our study: (1) we visualized spaced plant biomass yield for the 4 subpopulations and the 10 sites using an additive main effects and multiplicative interaction (AMMI) model to survey GxE interactions; (2) we estimated the genetic correlations among spaced plant biomass yield, fall height, and flowering time among regions and subpopulations; (3) we estimated the predictive ability of genomic prediction across environments by simulating a breeding program with genotypes evaluated at a random subset of sites (0–5); and (4) we determined the predictive ability of genomic prediction in a resource-limited breeding program minimizing field evaluation effort.

## Methods

### Field evaluations

Field data used for this analysis were previously described in Lovell *et al*. (2021) and Napier *et al*. (2022). To summarize, clones of a largely wild-collected diversity panel were established as spaced plants in a honeycomb pattern in 2018 at 10 sites spanning 17° of latitude (from Texas to South Dakota; Table 1). Genotypes were randomly assigned to sites with 0–2 replicates per site, with 53% of genotypes replicated at 8–10 sites and 36% replicated at 5 or fewer sites. The 3 primary traits (biomass, fall height, and flowering time) were measured at each site from 2019 to 2021. The plant biomass was measured (in grams) in October or November, depending on site. Whole plants were cut with a sickle bar mower and weighed using a hanging scale (OP-926, Optima Scale, Inc.). Dry weight adjustments for each sample were based on subsamples of approximately 500 g fresh material collected from each sample. Plants that were recorded as dead in the spring or summer received a biomass weight of 0 for that year. Flowering time was the ordinal day of year when 50% of panicles were undergoing anthesis for an individual plant. Height was measured as the mean panicle apex at the end of the season prior to harvest (in centimeters). To normalize biomass dry weight observations, a square root transformation of grams dry biomass was used and reported throughout this study. The phenotypic correlations in the mean biomass performance for each genotype are presented in Supplementary Fig. 1.

**Table 1.** Site locations, elevation, and soil type for each of the 10 field sites.

Site	Code	Region	Latitude	Longitude	Elevation (m)	Soil type
Kingsville, TX	KING	South	27.5498	−97.881	22	Cranell sandy clay loam
Austin, TX	PKLE	South	30.3839	−97.7293	235	Austin-Urban land complex
Temple, TX	TMPL	South	31.0433	−97.3494	182	Houston black clay
Overton, TX	OVTN	South	32.3029	−94.9794	138	Lilbert loamy fine sand
Stillwater, OK	STIL	Middle	35.9911	−97.0464	280	Teller fine sandy loam
Columbia, MO	CLMB	Middle	38.8969	−92.2178	268	Leonard silt loam
Lincoln, NE	LINC	North	41.1543	−96.4153	348	Filbert silt loam
Batavia, IL	FRMI	North	41.8367	−88.2396	225	Mundelein silt loam
Hickory Corners, MI	KBSM	Middle	42.4196	−85.3712	289	Kalamazoo loam
Brookings, SD	BRKG	North	44.3068	−96.6705	503	Brandt silty clay loam

### Molecular data

Marker data for the current study were based on the sequencing of tetraploid individuals presented in Lovell *et al*. (2021). Briefly, single nucleotide polymorphisms were based on deep (median depth 59) coverage using 2 × 150 bp paired-end PCR-free sequencers at HudsonAlpha Institute for Biotechnology and the Joint Genome Institute. Markers were filtered, retaining those with <20% missing data and minor allele frequency of >0.005. Missing markers were imputed to the population mean for that marker. This resulted in over 33 million markers. Switchgrass is an out-crossing heterozygous polyploid. Released cultivars are either tetraploid or octoploid. Tetraploids have disomic inheritance (Okada *et al*. 2010). Therefore, the realized relationship matrix between tetraploid genotypes was calculated using the A.mat function in the rrBLUP package (Endelman 2011). Subpopulation assignments and admixture were determined using STRUCTURE (Pritchard *et al*. 2000), as described in Lovell *et al* (2021).

### Objective 1: AMMI analysis

An AMMI model was used to visualize the relationships between experimental field sites and genetic subpopulations based on the spaced plant biomass yield (Crossa *et al*. 1991). For AMMI analysis, individuals, which were admixed, were removed (*n* = 566 remaining). Analysis was carried out using the *agricolae* R package (Mendiburu and Simon 2015). The model is


$$Y_{ij}=\;\mu +g_{i}+\;l_{j}+\;\overset{n}{\underset{k=1}{\sum }}\;\lambda_{k}\;\alpha_{ik}\;\gamma_{\;jk}+\;E_{ij}$$

where $Y_{ij}$ is the mean spaced plant biomass yield for the *i*th individual in the *j*th environment; *μ* is the grand mean; $g_{i}$ is the individual deviation from the grand mean; $l_{j}$ is the site deviation from the grand mean; $\;\gamma_{\;jk}\;$ is the eigenvalue value of the principal component analysis axis *k*; $\alpha_{ik}$ and $\;\gamma_{\;jk}$ are the principal component scores for of individuals and sites for axis *k*, respectively; *n* is the number of principal components used in the model; and $E_{ij}$ is the residual error. The model was run and visualized in 2 ways. First, the $g_{i}$ term aggregated all observations into the 4 subpopulations to visualize the mean subpopulation GxE within the data set. Next, an AMMI model was used to visualize GxE at the individual genotype level.

### Objective 2: surrogate trait utility across regions and subpopulations in multitrait models

It is assumed that different regions and subpopulations will have different surrogate trait relationships to yield. For the multitrait analysis, the data set was subdivided into 4 subpopulations (Gulf, Texas, Coastal, and Midwest; after Lovell *et al*. 2021) and 3 breeding regions (South, Middle, and North; Vogel *et al*. 2005; Casler 2012). The data set was divided into regions for this to minimize the GxE interactions present in the complete data set.

Within each region–subpopulation combination, single-trait and multitrait mixed models were compared to determine the potential benefit of multitrait models and determine the genetic correlations among flowering time, fall height, and biomass. The following model was run as both single-trait and multitrait for each region–subpopulation combination:


$$y_{ijk}=\mu +s_{i}+g_{j}+\varepsilon_{ijk}$$

For multitrait models, y*_ijk_* is a vector with length *n x t* (*n* genotypes and *t* traits) containing a combination of mean biomass, fall height, and flowering time for individuals at a site (measured across 3 years). Multitrait models estimated an unstructured variance–covariance between traits. For single-trait models, y*_ijk_* is solely the mean biomass for an individual at a site measured across 3 years. In both models, *μ* is the model intercept; *s_i_* is the fixed effect of the *i*th site; *g_j_* is the random effect of the *j*th genotype; and *ε_ijk_* denotes residuals [i.i.d. ∼*N*(0, σ_e_^2^)]. Genotypic effect variance–covariance structures were estimated using marker relationships [*p_j_* ∼ MVN(0, **G** σ_g_^2^)]. The **G** matrix is the realized relationship matrix (described above). This model was solved using the R package “sommer” (R Core Team; Covarrubias-Pazaran 2016). The estimated covariance among traits was converted to genetic correlations between traits for each subpopulation and region combination. The above mixed model was also used to determine the variances and estimate the narrow sense heritability on a genotype difference basis for biomass in a multitrait and single-trait model (Cullis *et al*. 2006; Schmidt *et al*. 2019).

### Objective 3: GxE genomic prediction and CV for biomass yield

Here, we focused on simulating breeding programs where genotypes are evaluated in either no sites (CV1) or only some of the sites (CV2). Individuals that were evaluated at >5 sites were included in CV populations (Texas *n* = 119 out of 129; Coastal *n* = 147 out of 280; Midwest *n* = 67 out of 122; Gulf *n* = 51 out of 54). These highly replicated individuals were used since they have highly reliable observed breeding values among most sites. The Gulf subpopulation was dropped from further analysis due to insufficient number of unique individuals. Validation genotypes were randomly assigned into 1 of 8 subsets. To simulate sparse designs, model training data included a variable number of field observations of biomass for validation genotypes (either 0 sites, CV1, or 1–5 randomly selected sites, CV2). The model (described below) was repeated using a leave-one-subset-out strategy where field observations from 1 subset was omitted from the model and predicted. Each 8-fold leave-one-subset-out cycle was repeated 5 times (40 times total per subpopulation), and the mean predictive ability of replicates is reported. Predictive ability was based on correlations among the observed and predicted breeding values of validation individuals in all sites, which did not have training data observations for that individual.

For simplicity, the mean biomass performance at individual sites (*n* = 10) was modeled as correlated traits using a Bayesian multivariate Gaussian model using the MTM package (Pérez-Rodríguez and de los Campos 2022). A Bayesian model was used for prediction among sites because classic mixed models, such as in the sommer package, failed to converge when attempting to estimate the large number of covariances in the multisite unstructured matrix. The following equation was used for genomic prediction:


$$y_{ij}=\mu +g_{A\;}+\varepsilon_{ij}$$

where *μ* is the model intercept; *y_ij_* is the scaled phenotypic data with *i* equal to the number of sites; *j* is the number of individuals; **g**_A_ is the breeding value for switchgrass individuals at each site (or trait, see Objective 4, below) considered as random with **g**_A_ ∼*MVN*(0, ***T****_a_* ⨂ ***G***), where ***T****_a_* is the unstructured variance–covariance matrix among sites or traits; and ***G*** is the realized relationship matrix (described above). The ⨂ symbol denotes the Kronecker product, and *ε_ij_* is the residual vector with *ε_ij_*∼(0, **I**σ^2^*_ε_*). For each iteration, we assumed 25,000 Gibbs samples with a burn-in of 200 thinned of 5.

### Objective 4: genomic prediction in resource-limited scenarios

A multitrait, multienvironment model was used to estimate the predictive ability of 6 potential resource-efficient evaluation strategies for new untested genotypes. Generally, these scenarios assume a core training data set of biomass and yield surrogate measures and will attempt to provide the validation population with the minimum field data to obtain a useful prediction. This will use the same 8-fold CV scheme described above. For each scenario, the validation population (subset of individuals replicated at >5 sites) only has the following data included in the training data set: (1) no phenotypic observations to represent an untested genotype (CV1); (2) 1 site observation of flowering time; (3) 1 site observation of flowering time and height; (4) 1 site observation of fall height; (5) 1 site observation of biomass (CV2); and (6) 5 site observations of biomass (CV2).

For multitrait, multisite predictions, flowering time and fall height were assumed to have negligible GxE relative to biomass yield. These surrogates were included as an additional correlated trait (equivalent to an additional environment) in the multienvironment model from Objective 3. Surrogates would often be measured at a single site in a realistic breeding program. Therefore, the mean flowering time and fall height observed at both KBSM and CLMB were combined to provide a general measure for the 2 surrogates. These 2 sites were used solely because they collectively include observations for 553 of 585 switchgrass individuals for flowering and 554 of 585 switchgrass individuals for fall height.

## Results

### AMMI analysis

**Fig. 1.**
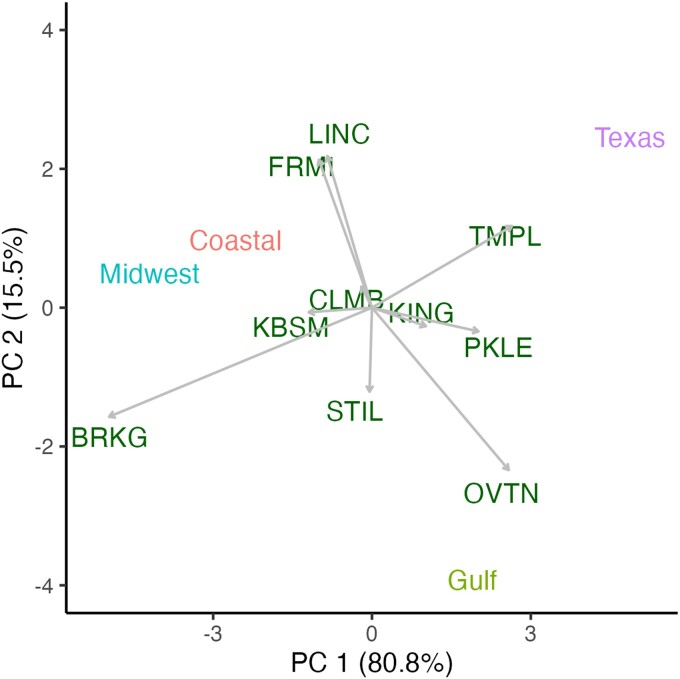
AMMI model biplot showing the relationship among 10 sites and the mean biomass performance of the 4 major subpopulations of switchgrass (*Panicum virgatum*). Scores near 0 indicate less of a GxE interaction. Subpopulations that occur near field sites in the biplot indicate overperformance of those subpopulations in those sites.

**Fig. 2.**
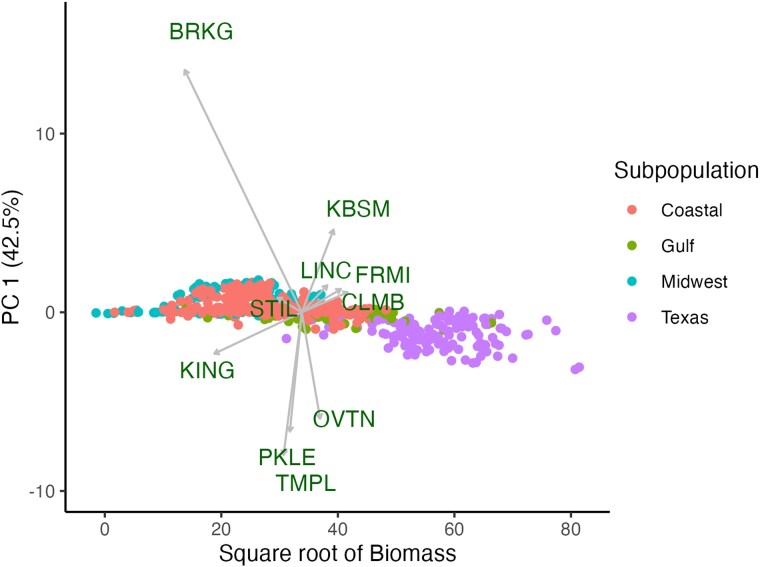
Plot showing the mean biomass performance across environments (grams plant^−1^ year^−1^; square root transformed; *x*-axis) and the first principal component (PC1) from an AMMI model summarizing biomass variation among 10 sites (*y*-axis).

In interpreting the results, it is critical to keep in mind that the germplasm used in this study represents a diverse collection of population accessions, which is a major deviation from the classic quantitative genetic assumption of a panmictic breeding population. To partially control for this high genetic diversity, we partitioned the diversity panel into 4 switchgrass subpopulations, which are potential germplasm sources for future breeding programs. This division into 4 subpopulations is different from the approach that Lovell *et al*. (2021) took, with the assignment of all genotypes to only 3 subpopulations. The division between Gulf and Texas subpopulations had been informally observed in prior genomic analysis, but Texas individuals were generally grouped within the Gulf subpopulation. During analysis for this study, the AMMI analysis made the distinction between the Gulf and Texas subpopulations apparent based on field performance (Figs. 1 and 2).

Broadly, the 4 subpopulations tended to have superior performance near their collection regions (Figs. 1 and 2). The Gulf and Texas subpopulations performed well in southern sites, while the Midwest and Coastal subpopulations performed well in northern sites. When comparing the aggregate performance of the 4 breeding groups, the first AMMI principal component accounted for 80.8% of the GxE interactions (Fig. 1) and generally aligned with the latitude of the common gardens with winter tolerant subpopulations (Midwest and Coastal) overperforming at northern sites. The second principal component explained 15.5% of the variance and accounted for the difference in the performances of the Texas and Gulf subpopulations. Differentiation among sites in the second principal component did not follow an obvious geographic or environmental pattern.

On an individual basis, the AMMI first principal component (42.5% of variance) again aligned with the latitude of the sites (Fig. 2) and broadly predicted similar patterns within both subpopulations and sites. The second and third principal components accounted for 13.6 and 9.7% of the GxE variance, respectively. Relative to the variance observed among sites, there was also a large degree of genotypic variance for biomass that was not influenced by environment or by subpopulation (Fig. 2).

### Multitrait models

Across all region–subpopulation combinations, the mean of multitrait model estimates of biomass heritability was 0.69, while single-trait models (using only biomass yield) had an estimated heritability of 0.59 (Supplementary Table 1). Multitrait models, which assumed unstructured covariance between height, flowering time and biomass, indicated generally positive genetic correlations between these traits across all region–subpopulation combinations (Supplementary Fig. 2). The mean genetic correlation between fall height and biomass was 0.85. The mean genetic correlation between flowering and biomass was 0.45. The mean genetic correlation between flowering and height was 0.31. The only negative relationship observed was within the Midwest subpopulation grown in the South region, which had a −0.39 genetic correlation between flowering time and height and overlapped with the lowest (0.15) correlation between flowering time and biomass.

It should be noted that certain region–subpopulation combinations are not valuable or informative from a practical agronomic perspective (Supplementary Fig. 2). Specifically, the relationships among traits for the Midwest subpopulation in the south are not useful because the subpopulation is maladapted to these sites and has high mortality. Furthermore, there does not appear to be any value in breeding northern germplasm to be grown in southern regions, as it is generally lower yielding than locally adapted southern germplasm. While not all combinations of genotypes and field sites are relevant from an agronomic focus, these combinations provide insights into what drives the geographic ranges of the different ecotypes of switchgrass.

### Genomic prediction

**Fig. 3.**
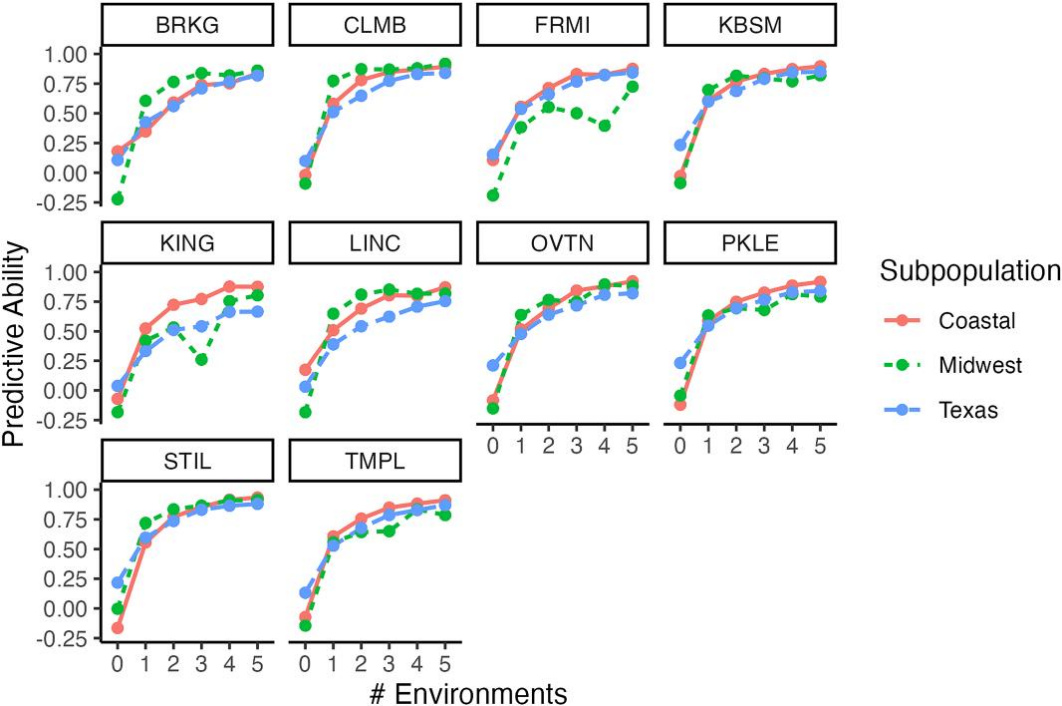
Mean predictive ability across 10 sites for 3 switchgrass subpopulations based on CV of individuals that had biomass performance measured at 0, 1, 2, 3, 4, and 5 sites (# environments) included in the training data. Predictive ability is the correlations among predicted and observed performances of omitted individuals for a given site. Point and line colors indicate the subpopulations.

Generally, the CV predictive ability of biomass performance was low in CV1 (−0.02) and reached a plateau once >2 sites were observed for most subpopulation–site combinations (>0.60; CV2), where additional sites resulted in minor improvements in predictive ability (Fig. 3). There was little variation in the predictive ability due to region, subpopulation, or site. In the CV1 scenario, the Texas subpopulation had a greater predictive ability (0.15) relative to the Coastal (0.01) and Midwest subpopulations (−0.13). However, after a single site observation was included (CV2), the trend in the mean predictive ability reversed (Midwest: 0.61; Coastal: 0.54; Texas: 0.49).

**Fig. 4.**
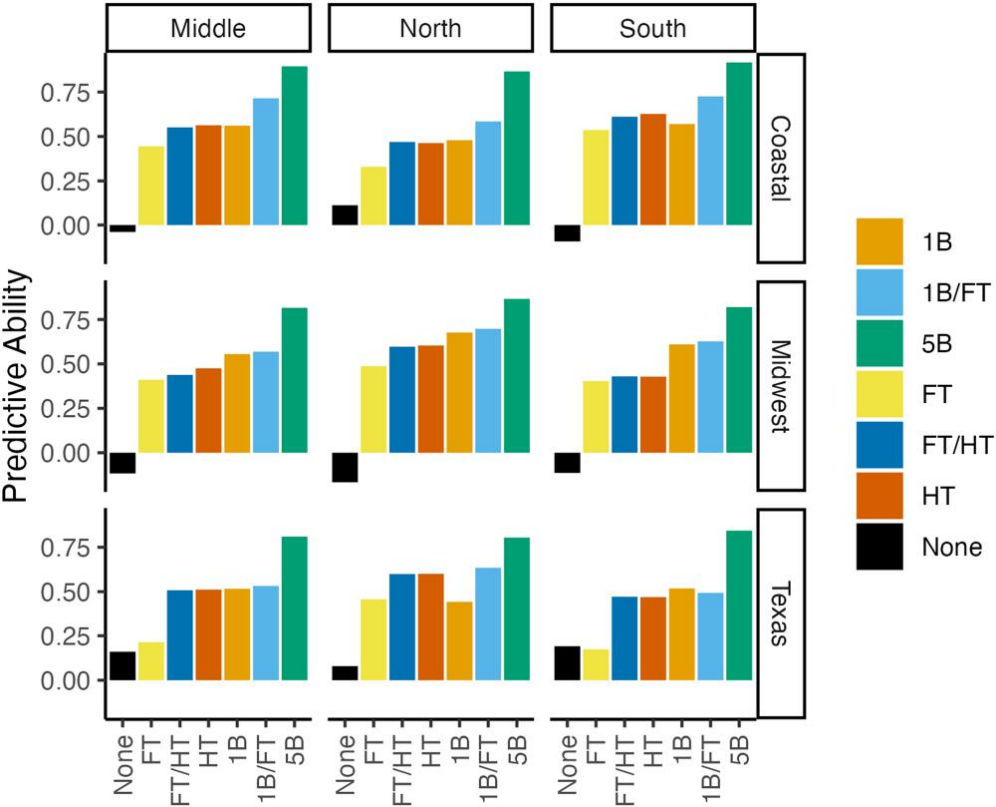
Predictive ability across 10 sites within 3 regions for 3 switchgrass subpopulations using models that simulate a range of the field data collection effort for the validation population. Field observations for validation individuals included the following: no observations (CV1: None), 1 site flowering time measurement (FT), 1 fall height and 1 flowering time measurement (FT/HT), 1 site fall height measurement (HT), 1 site biomass measurement (B1), 1 flowering time measurement and 1 biomass measurement (1B/FT) biomass evaluations at 5 sites (5B). Predictive ability is the correlation among the predicted and observed performances of omitted individuals in unknown sites. Bar colors indicate the different levels of field observation effort.

The Objective 4 multitrait prediction model found a range of predictive abilities (Fig. 4). In order to increase the predictive ability, the models included the following data for validation genotypes: no field data (CV1; −0.02); 1 site measurement of flowering time (0.39); 1 site measurement of flowering time and height (0.51); 1 site measurement of fall height (0.52); 1 measurement of biomass (0.56); 1 site measurement of biomass; 1 measurement of flowering time (0.62); and 5 site measurements of biomass (0.84). Model performance did not strongly differ among regions and subpopulations (Fig. 4).

## Discussion

### Spaced plant biomass performance varies across a north–south axis

In this study, we analyzed a large switchgrass diversity panel across multiple environments with a focus on agronomics and plant breeding. Prior studies have reported strong GxE variation in this data set (Lovell *et al*. 2021; Napier *et al*. 2022) and in independent switchgrass studies across this north–south transect of field sites (Lowry *et al*. 2019).

Overall, we clustered our 10 field sites into 3 geographic regions to be informative when thinking about the target regions for switchgrass breeding programs, but that information could be successfully shared across all 10 sites (Figs. 1 and 3). This study consists of 10 common garden sites aligned along a north–south transect with relatively little east–west contrast. Therefore, it is unsurprising that the first principal component for both AMMI analyses aligned with latitude and accounted for >40% of the GxE variation (Figs. 1 and 2). One deviation from this trend was the southernmost site, KING, which occurred closer to the origin relative to the other southern sites (Figs. 1 and 2). This site has many distinct environmental characteristics that could explain this deviation.

### Yield surrogates are useful across all regions and subpopulations

Flowering time and height measurements are valuable yield surrogates across all regions and subpopulations (Fig. 4; Supplementary Fig. 2). The mean genetic correlations between biomass and flowering time tended to decrease in southern regions (North: 0.52; Middle: 0.47; South: 0.34) and with later flowering germplasm (Midwest: 0.56; Coastal: 0.41, Gulf: 0.52; Texas: 0.30). However, all biomass–flowering time correlations remained positive. This not only reinforces prior studies, which reported that the selection for late flowering is a useful surrogate for improving the biomass yield (Price and Casler 2014; Casler 2020; Tilhou and Casler 2022a), but also suggests that selection for late flowering to increase biomass will be most effective for production in the North.

Interestingly, fall height had a greater genetic correlation with spaced plant biomass relative to flowering time (0.85 vs 0.45). This is promising since fall height is easier to measure compared to flowering time. Previously, flowering time was assumed to be more useful than fall height because flowering time is less influenced by within-field variability (unpublished data; Price and Casler 2014; Casler 2020; Tilhou and Casler 2022a, 2022b). One caveat to the current analysis is that flowering time, height, and biomass were measured on the same plants. Therefore, correlated errors due to field heterogeneity may inflate the estimated genetic correlation between surrogates and biomass. Previously, Price and Casler (2014) reported a strong positive genetic correlation between height and sward biomass, but that selection for spaced plant height was insufficient to improve biomass. Interestingly, they reported that selection for late flowering successfully improved both height and biomass. Similarly, Van Esbroeck *et al*. (1998) reported an improvement in height by selection for flowering time in Texas using the southern Alamo switchgrass cultivar. The current study builds on these results and provides robust evidence that selection for some combination of late flowering and fall height can improve biomass production, even in southern regions or in programs where the germplasm is already late flowering (Supplementary Fig. 2; Fig. 4).

### Relating spaced plant and sward performance is challenging

One major caveat of our study is that all measurements were collected in spaced plant conditions, which differs greatly from what plants experience when grown in commercial-scale swards. The ability of spaced plant biomass to predict biomass performance in swards has varied greatly across studies. Within a breeding population, Casler and Ramstein (2018) reported a correlation of only 0.24 between spaced plant biomass and sward biomass. However, later studies found slightly stronger relationships (Casler 2021: >0.50; Tilhou and Casler 2022b: 0.58 and 0.27). The best analogy for spaced plant measurements is that these measure potential performance under ideal conditions. This is similar to ranking soccer (football) players based solely on their sprinting speed across an empty field. While this strategy would be sufficient to identify potentially elite players, it is clearly an oversimplification. Spaced plants have massively reduced above- and below-ground competition relative to a seeded sward. Therefore, maximizing productivity in commercial conditions will likely require additional traits, which may be region specific and include disease resistance, nutrient efficiency, or adaptation to soil conditions (Casler *et al*. 2019; Songsomboon *et al*. 2019; VanWallendael *et al*. 2020).

Another characteristic of spaced plant biomass evaluations is that they amplify genetic variation in a breeding population (Casler 2021). For example, this data set includes elite individuals with 100-fold greater mean spaced plant biomass relative to the smallest individuals (Fig. 1). This is partially due to the study's goal of representing the full diversity of switchgrass genetic diversity, but the effect also occurs in breeding panels. For example, Casler and Ramstein (2018) reported correlations among spaced plant biomass and sward biomass where spaced plant biomass ranged from approximately 0.25 to 0.90 kg plant^−1^, depicting a difference in performance of >200%. However, the same genotypes only had a range of 13.5–15.5 Mg ha^−1^ biomass yield in sward plots, yielding a potential difference of 15%. While this indicates that there is not a proportional relationship between sward yield and space plant biomass potential, the amplified genotypic variance may improve the ability to detect superior individuals in spaced plant evaluations.

### Implications for breeding programs

Obtaining reliable breeding values for sward biomass of switchgrass genotypes requires slow (3–5 years) and relatively expensive field scale trials. Since height, flowering time, and spaced plant biomass are all linked and have some positive genetic relationship to sward yield, a rapid multitrait selection scheme will often be a more cost-effective alternative. A surrogate-based selection scheme could require 2–3-year cycles using field evaluations or even shorter cycles using genomic prediction (Tilhou and Casler 2022a). Additional traits could be used to further improve prediction of sward biomass yield. For example, tiller count was collected in this study and positively correlated to biomass in spaced plants (data not presented), but this trait was not included because of multiple reports of a negative or neutral relationship to biomass yield in sward plots (Smart 2001; Price and Casler 2014; Sykes *et al*. 2017; Tilhou and Casler 2022b). Mitchell *et al*. (2023) reported that switchgrass and big bluestem seedlings selected for multiple tillers had increased yield over single tiller seedlings in space-planted nurseries, but those yield increases did not translate to increased yields when grown in swards. Spring green-up is another potential surrogate, which was not included in this analysis, but may have predictive value in the southern United States (Ali *et al*. 2019).

The strong CV predictive ability for biomass among the 10 sites of this study underscores the strength of the sparse experimental design. With only 1 site observation, predictive ability for biomass yield was >50% of the maximum and predictive ability and rapidly reached a plateau with increasing observations (Fig. 3). Predictive ability for individuals with no phenotypic data in the model was consistently near 0, which is reasonable given the small training population size and broad genetic distances within each of these subpopulations. Therefore, evaluations of either spaced plant biomass, height, or flowering time only need to occur at a small number of sites. In the models, which minimized field data collection (Fig. 4), there was a steady increase in predictive ability with increasing field measurement effort. Flowering time and height measurements require fewer resources relative to biomass harvests in spaced plants. When combined with sparse experimental designs, these low-input surrogate measures can drastically reduce the field evaluation expenses of a breeding program, with the caveat that an affordable and rapid sequencing platform needs to be available.

In resource-limited breeding programs, regular large-scale sward evaluations may often not be practical. Therefore, a breeding program, which relies heavily on spaced plant surrogates, will be the pragmatic choice. Casler (2020) reported a successful example of a low-cost, rapid selection scheme that evaluated individuals in only 1 environment for flowering time and was able to improve biomass in swards across a wide range of environments (mean of 2.3% gain in sward yield year^−1^). Similarly, Missaoui *et al*. (2005) also reported sward biomass yield improvement at multiple sites based on spaced plant biomass selections at a single site (mean of 1.6% gain in sward yield year^−1^). Intense selection based solely on surrogates, therefore, may have the potential to rapidly increase the switchgrass performance. This study highlights that it may provide adaptive benefits across a wide geographic region.

## Supplementary Material

jkae159_Supplementary_Data

## Data Availability

Complete sequencing data for the switchgrass panel are available at the NIH NCBI Sequence Read Archive (SRP258586). In addition, the realized relationship matrix, mean field performance, subset of SNPs, and example code files are provided at Dryad Digital Repository (https://doi.org/10.5061/dryad.s4mw6m9cs). Supplemental material available at G3 online.
