# Supplementary material for: Genomic prediction of regional-scale performance in switchgrass (Panicum virgatum) by accounting for genotype-by-environment variation and yield surrogate traits
Source: G3 (Bethesda). 2024 Jul 19;14(10):jkae159. doi: 10.1093/g3journal/jkae159 (PMC11457067; doi:10.1093/g3journal/jkae159)
Supplement: jkae159_Supplementary_Data [file jkae159_supplementary_data.docx]

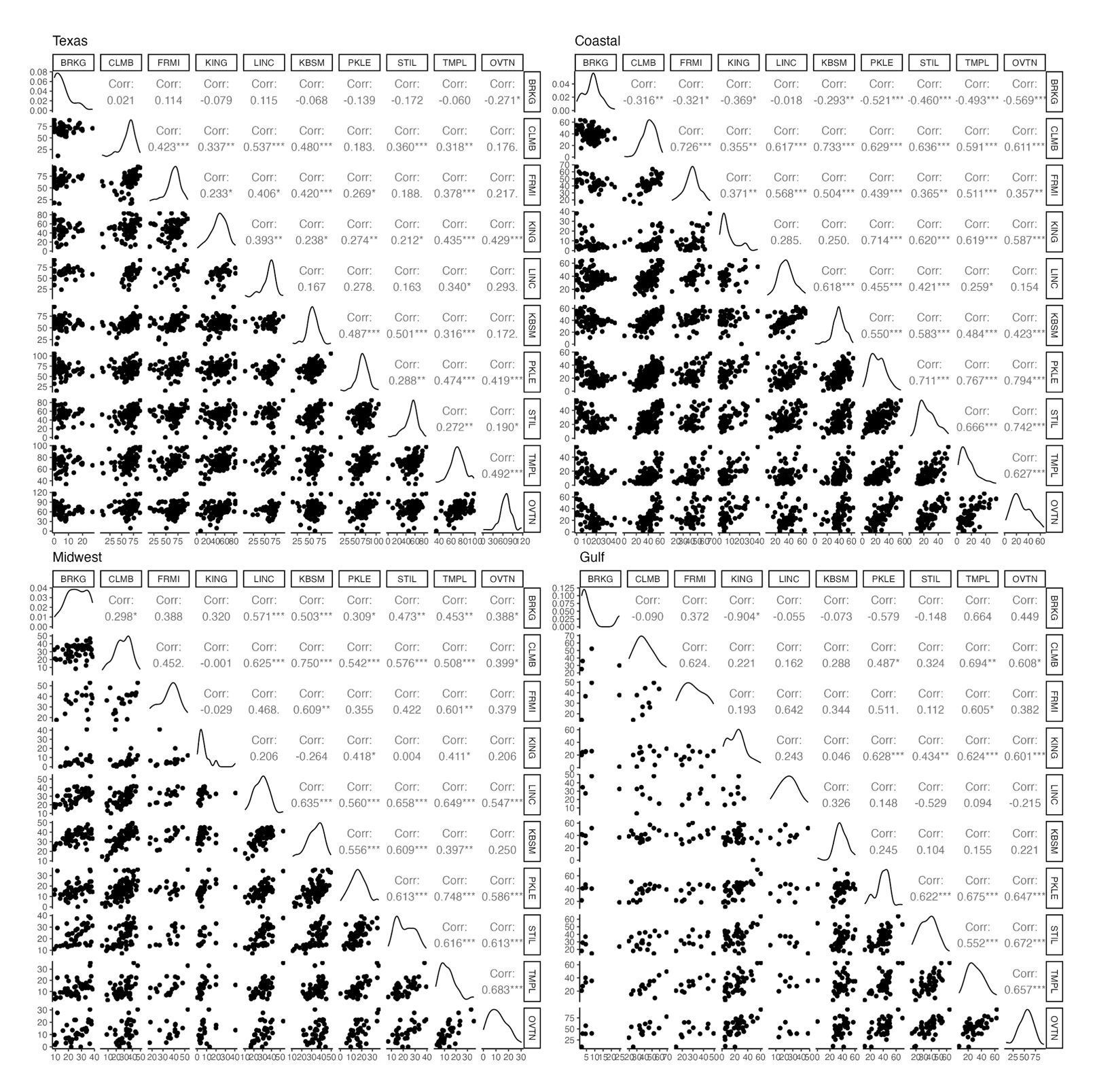


Supplementary Figure 1: Pairwise phenotypic correlations among mean biomass yield (square root adjusted) of genotypes from four switchgrass subpopulations among the 10 environments across 3 years. Diagonals indicate the distribution of mean biomass yield. The values above the diagonal are Pearson correlation coefficient and below diagonals are scatterplots of genotypes grown at both sites.


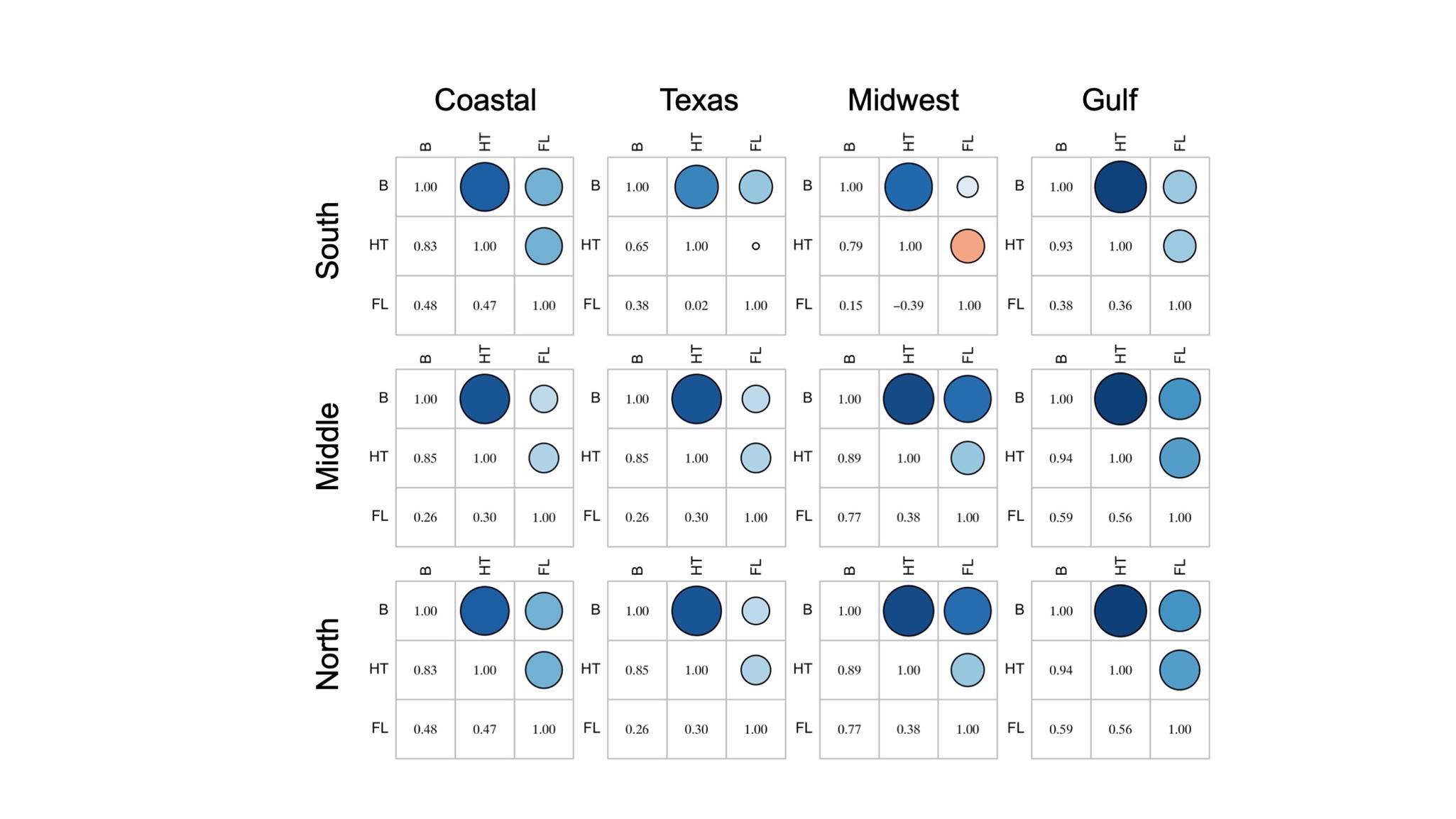


Supplementary Figure 2: Genetic correlations for three traits (B: Biomass, HT: Height, FL: Flowering Time) within the four primary subpopulations of switchgrass across three regions. Circle size (above diagonal) and shade indicate the strength and direction of correlation (blue represents positive, and red represents negative correlations).

Supplementary Table 1: Additive genetic variance (σ_A_^2^), residual genetic variance (σ_R_^2^) and heritability (H^2^_cullis_) for multi-trait and single trait (ST) models for four switchgrass breeding populations across three growing regions.

| **Region** | **Subpopulation** | **Trait** | **σ_A_^2^** | **σ_R_^2^** | **H^2^_Cullis_** |
| --- | --- | --- | --- | --- | --- |
| Middle | Coastal | Biomass | 27 | 36 | 0.57 |
| Middle | Coastal | Height | 295 | 236 | 0.61 |
| Middle | Coastal | Flowering | 135 | 111 | 0.58 |
| Middle | Coastal | Biomass (ST) | 26 | 39 | 0.47 |
| South | Coastal | Biomass | 30 | 31 | 0.75 |
| South | Coastal | Height | 371 | 231 | 0.76 |
| South | Coastal | Flowering | 173 | 138 | 0.66 |
| South | Coastal | Biomass (ST) | 31 | 33 | 0.60 |
| North | Coastal | Biomass | 25 | 34 | 0.61 |
| North | Coastal | Height | 218 | 155 | 0.68 |
| North | Coastal | Flowering | 92 | 19 | 0.80 |
| North | Coastal | Biomass (ST) | 25 | 35 | 0.51 |
| Middle | Gulf | Biomass | 32 | 86 | 0.76 |
| Middle | Gulf | Height | 349 | 316 | 0.80 |
| Middle | Gulf | Flowering | 168 | 346 | 0.64 |
| Middle | Gulf | Biomass (ST) | 36 | 89 | 0.57 |
| South | Gulf | Biomass | 105 | 58 | 0.90 |
| South | Gulf | Height | 750 | 214 | 0.92 |
| South | Gulf | Flowering | 272 | 229 | 0.78 |
| South | Gulf | Biomass (ST) | 101 | 59 | 0.83 |
| North | Gulf | Biomass | 54 | 48 | 0.31 |
| North | Gulf | Height | 399 | 186 | 0.33 |
| North | Gulf | Flowering | 254 | 22 | 0.38 |
| North | Gulf | Biomass (ST) | 44 | 58 | 0.19 |
| Middle | Midwest | Biomass | 25 | 30 | 0.66 |
| Middle | Midwest | Height | 222 | 270 | 0.60 |
| Middle | Midwest | Flowering | 113 | 123 | 0.58 |
| Middle | Midwest | Biomass (ST) | 25 | 30 | 0.52 |
| South | Midwest | Biomass | 31 | 11 | 0.78 |
| South | Midwest | Height | 274 | 173 | 0.74 |
| South | Midwest | Flowering | 53 | 206 | 0.43 |
| South | Midwest | Biomass (ST) | 29 | 14 | 0.72 |
| North | Midwest | Biomass | 41 | 21 | 0.76 |
| North | Midwest | Height | 288 | 61 | 0.82 |
| North | Midwest | Flowering | 109 | 20 | 0.82 |
| North | Midwest | Biomass (ST) | 41 | 22 | 0.70 |
| Middle | Texas | Biomass | 49 | 84 | 0.77 |
| Middle | Texas | Height | 246 | 217 | 0.83 |
| Middle | Texas | Flowering | 77 | 205 | 0.59 |
| Middle | Texas | Biomass (ST) | 48 | 91 | 0.66 |
| South | Texas | Biomass | 64 | 83 | 0.75 |
| South | Texas | Height | 248 | 192 | 0.80 |
| South | Texas | Flowering | 192 | 179 | 0.76 |
| South | Texas | Biomass (ST) | 72 | 89 | 0.70 |
| North | Texas | Biomass | 72 | 61 | 0.71 |
| North | Texas | Height | 354 | 185 | 0.73 |
| North | Texas | Flowering | 83 | 29 | 0.73 |
| North | Texas | Biomass (ST) | 65 | 64 | 0.57 |
